# Supplementary material for: Dysfunctional Pro1 leads to female sterility in rice blast fungi
Source: iScience. 2023 Jun 14;26(7):107020. doi: 10.1016/j.isci.2023.107020 (PMC10320130; doi:10.1016/j.isci.2023.107020)
Supplement: Document S1. Figures S1–S17 and Table S1 [file mmc1.pdf]

iScience, Volume *26*

Supplemental information

Dysfunctional Pro1 leads to female sterility in rice blast fungi

Momotaka Uchida, Takahiro Konishi, Ayaka Fujigasaki, Kohtetsu Kita, Tsutomu Arie, Tohru Teraoka, Yasukazu Kanda, Masaki Mori, Takayuki Arazoe, and Takashi Kamakura

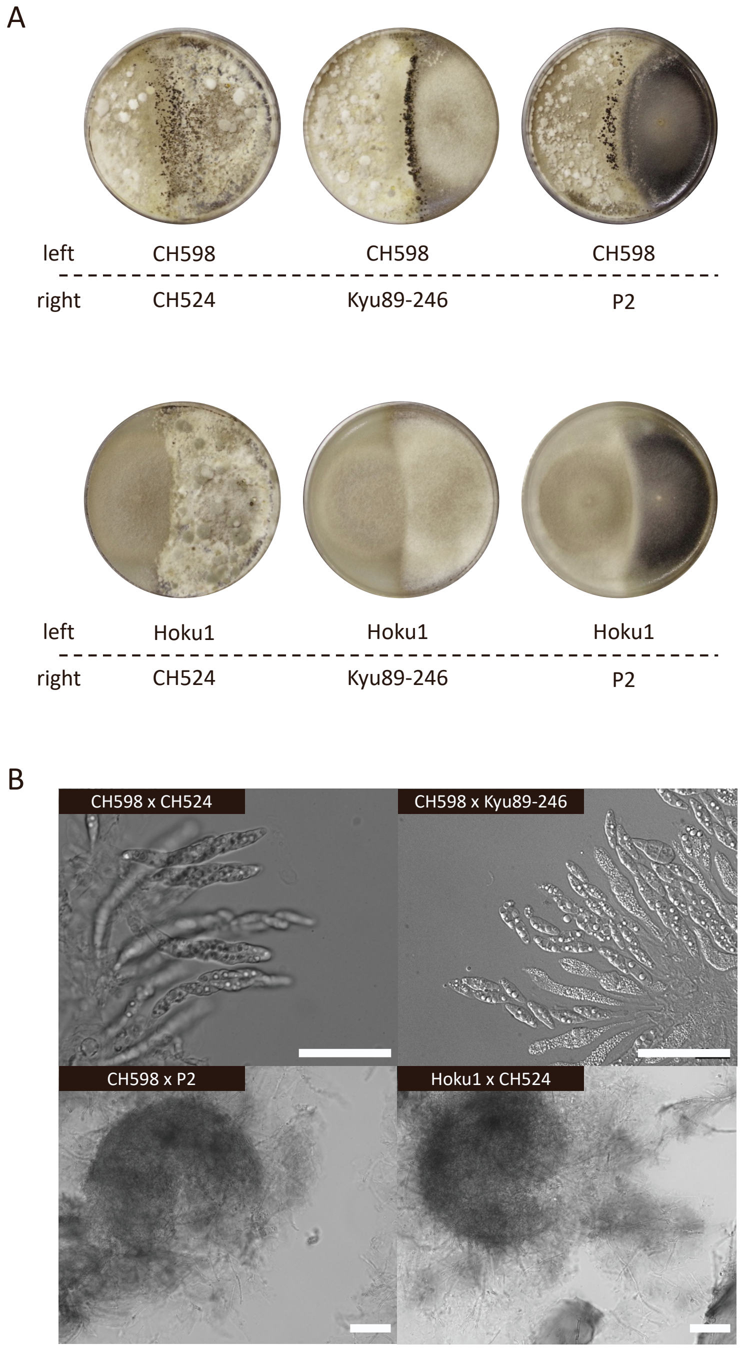

Fig. S1. Mating capability of six field isolates of *Pyricularia oryzae*, related to Fig. 1. (**A**) Perithecia developed by crossing CH598×CH524, CH598×Kyu89-246, CH598×P2, and Hoku-1×CH524. Hoku-1, Kyu89-246, and P2 did not develop perithecia. (**B**) Asci and ascospores developed by crossing CH598×CH524 and CH598×Kyu89-246. Perithecia contained no mature asci or ascospores between CH598×P2 nor Hoku-1×CH524. Scale bar = 50 µm.

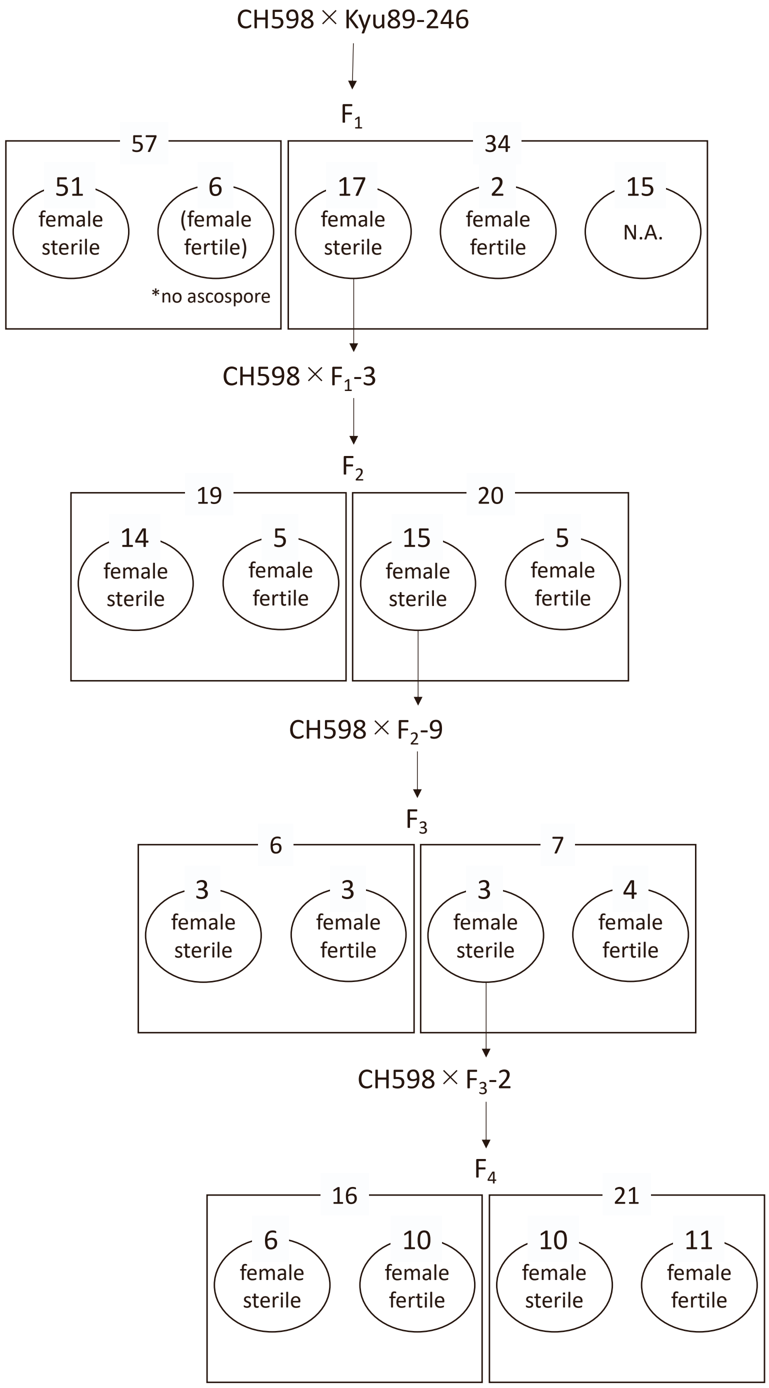

Fig. S2. Schematic diagram and the number of progenies obtained, related to Fig. 1. Female-sterile progenies in each generation were crossed with CH598, and the mating-type and female fertility of the progenies were examined. N.A., not assessed.

Gene locus	70-15 Chromosome	Female fertile contig	Female sterile contig	Amino acid substitution	Annotation
MGG_00791	#5	#180	#261	H201Y	Lactamase B domain-containing protein
MGG_00779	#5	#180	#261	V125G	choline dehydrogenase
MGG_00771	#5	#180	#261	G1343V	5-oxoprolinase
MGG_11498	#5	#180	#261	D121E	hypothetical protein
MGG_00747	#5	#143	#118	P38L	palmitoyltransferase PFA4
MGG_17384	#5	#143	#118	V166F	hypothetical protein
MGG_00722	#5	#143	#118	G604R	hypothetical protein
MGG_00706	#5	#143	#118	P197L	DNA polymerase subunit alpha B
MGG_00693	#5	#143	#118	T340S	hypothetical protein
MGG_11512	#5	#143	#118	Q203E	hypothetical protein
MGG_17398	#5	#16	#199 and #128	G164V	hypothetical protein

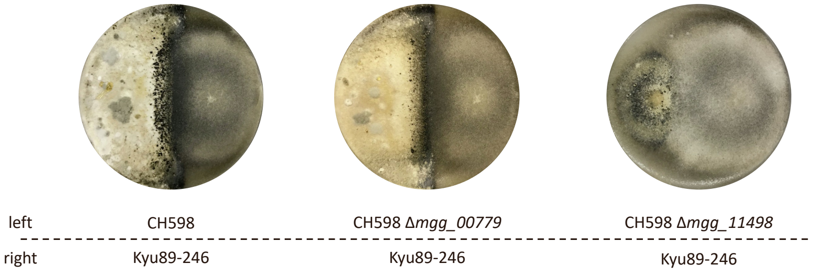

Fig. S3. Candidate genes involved in loss of female fertility in the FS1 region, related to Fig. 1. The candidate genes containing amino acid substitutions were listed in the upper table. The two genes were related to perithecium formation (shaded in orange in the upper table and lower pictures) and the three genes (colored with grey) were predicted to be lethal genes.

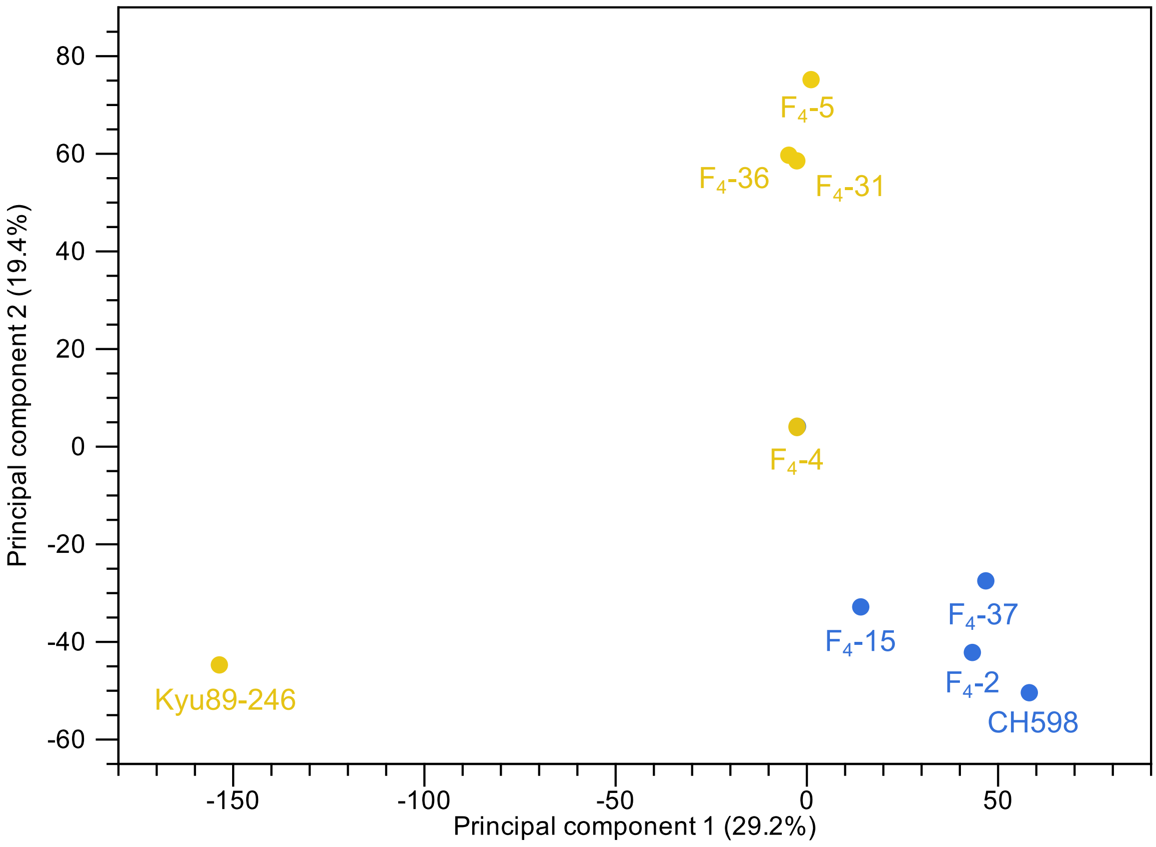

Fig. S4. Principal component analysis of RNA-seq data including F_4-4, related to Fig. 4.

Female-sterile-evolved F_4-4 showed relatively closer expression patterns to female-fertile strains.

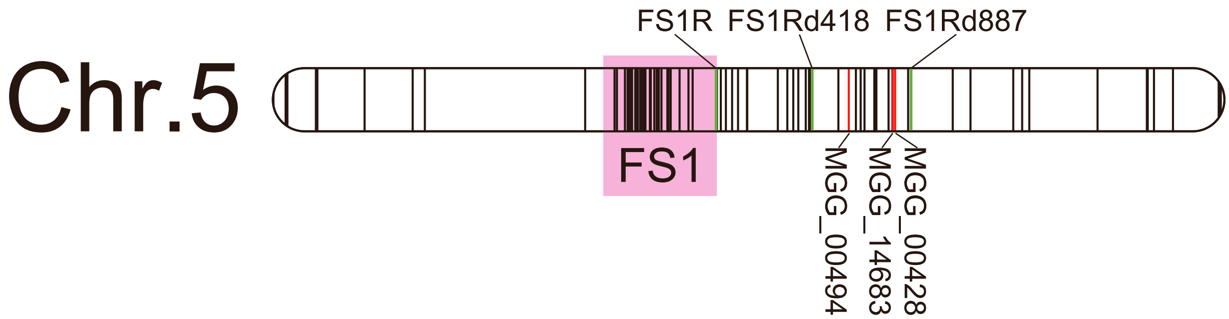

Fig. S5. Refined mapping of nucleotide substitutions, related to Fig. 1 and 2. The reads of female-fertile progenies were re-aligned to the *de novo* assembled genome of female-sterile progenies, and the detected substitutions were mapped to the reference genome.

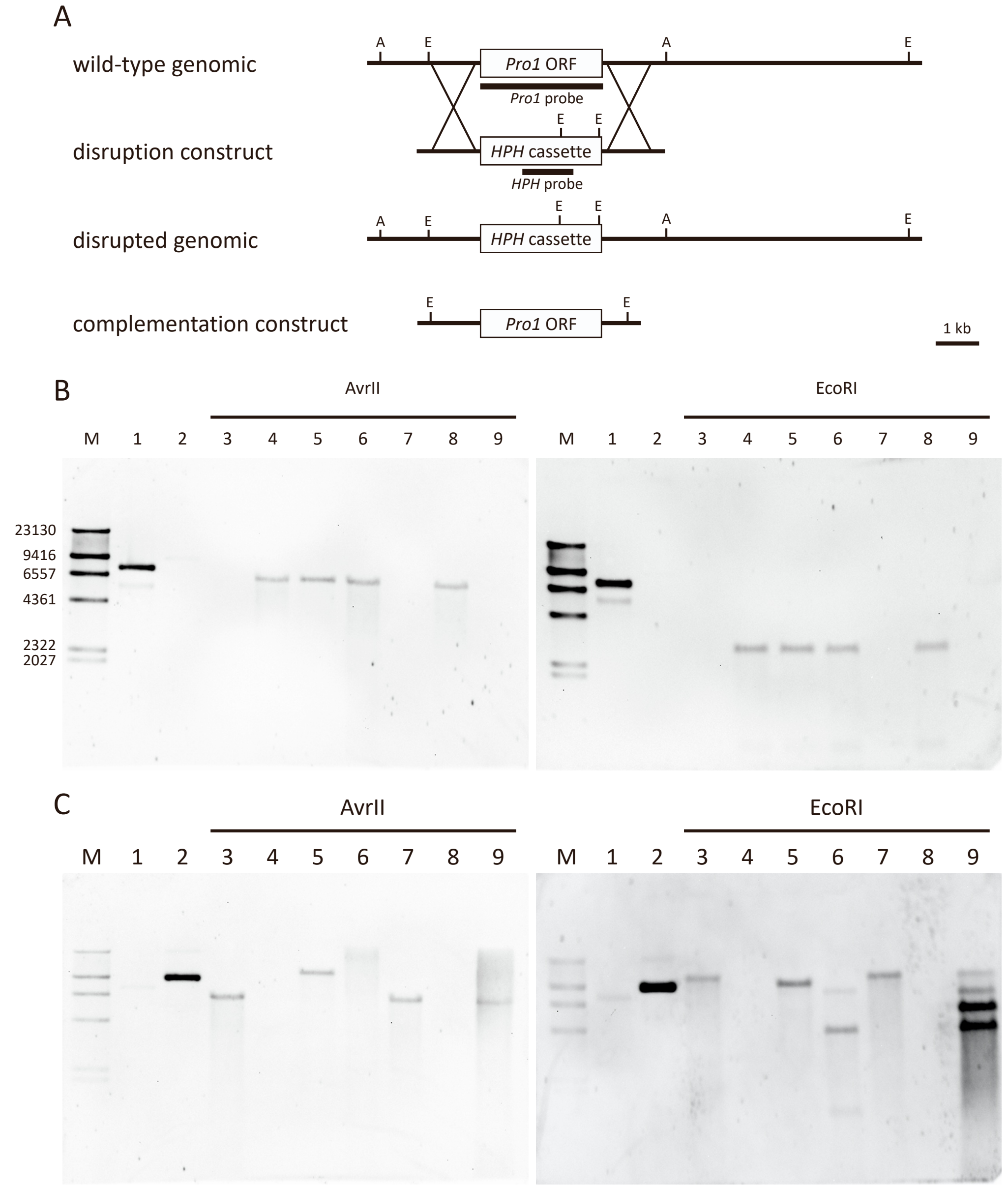

Fig. S6. Verification of transformants by Southern blotting, related to Fig. 3. (**A**) The physical maps of the plasmid constructs and genomic region surrounding the *Pro1* gene. DIG-labelled probes carrying the sequence of (**B**) *HPH* ORF and (**C**) *Pro1* ORF were individually detected. M, DIG-labelled λHindIII; lane 1, disruption construct (linearized by XbaI); lane 2, complementation construct (linearized by AflII); lane 3-9, CH598 wild type, CH598 Δ*pro1*, CH598 Δ*pro1*/*Pro1*^{CH598}, CH598 Δ*pro1*/*Pro1*$^{Kyu89\text{-}246}$, Kyu89-246 wild type, Kyu89-246 Δ*pro1*, Kyu89-246 *Pro1*^{CH598}, respectively.

```
P. oryzae Pro1             MSTISPNLQANLATASPVPKMAVASATKTKPAKAAAATANGTSNKKSQMHRRSRTGCYTC
S. macrospora Pro-1        --------------------MTTTTTTKTKA--TAKAGTNAAPKQKTQMHRRSRTGCYTC
N. crassa Adv-1            MSTQSPNHHEDITKTSSVNMTTTTTTTKTKA--AAKAGTNAAPKQKTQMHRRSRTGCYTC
                                               :.:::****.  :* * :*.:.::*:**************

P. oryzae Pro1             RLRRKKCDEGQHVCSACKHLGLVCEYKRPHWWSNNEARRRHKDDIKTIIKKKKLAEKGAH
S. macrospora Pro-1        RLRRKKCDEGSPMCTACKHLGLQCEYKRPMWWSNNDLRRKHKDEIKMIIKRKKLSEKSSH
N. crassa Adv-1            RLRRKKCDEGSPMCTACKHLGLCCEYKRPMWWSNNDARRKQKDEIKMIIKRKKLSEKSSH
                           **********. :*:******* ****** *****: **.:**:** ***.***:**.:*

P. oryzae Pro1             AVQTGMSSPPGLSHSIPSLPTSATFSDPLDRTRSASIDSHFSFNFNSPPTTCSEYGAF-T
S. macrospora Pro-1        TIQTSINTPPGLSH---SLPTSATFSGPLDRNRSASIDSHFGFNFNS-PHNGQDFTPFAT
N. crassa Adv-1            TIQTSINTPPGLSH---SLPTSATFSDPLDRNRSASIDSHFGFNFNS-PQHGQDFAAFAT
                           ::**.:.:******   *********.****.*********.***** *   .:: .* *

P. oryzae Pro1             PQLH--PDFMFTGPYASPYEIDVKTERQMFVNDVPTLRESTTSTFSTCYTPPPPGTTLPS
S. macrospora Pro-1        PQININGEFIFP-PFS-PYEIDMKTERQIFINDVPTLRESTVSTFSTYQTPPPPGTILPS
N. crassa Adv-1            PQIHVNGEYMFP-PFS-PYEIDMKTERQIFINDIPTLRESTVSTFSTYQTPPPPGTILPS
                           **::   :::*. *:: *****:*****:*:**:*******.*****  ******* ***

P. oryzae Pro1             FPLEGEWTEQVCSERRESFTEETLNVNFFDFAHGRSMQSRQVAIELDEGDQRLLDHFVRH
S. macrospora Pro-1        FPLEGEWTEQVFSERRESLTEETFNANFFDFACDPALASSQVAVELDEGDQKLFDHFVQH
N. crassa Adv-1            FPLEGEWTEQVFSERRESLTEETFNANFFDFACDPAMASSQVAIELDDGDQKLLDHFVQH
                           *********** ******:****:*.****** . :: * ***:***:***.*:****.*

P. oryzae Pro1             VLPTIFPILESNQHGSVSSDLILPALQSNKGYLHCCLSIAAQHLKAATGSQSEDMDADIM
S. macrospora Pro-1        VLPTIFPILESNQHGSISSELILPSLANNKGYLHCCLSIAAQHFKSTTGIQSEEIDNDIM
N. crassa Adv-1            VLPTIFPILESNQHGSISSELVLPSLANNKGYLHCCLSIAAQHFKSTMGIQNEEIDNDIM
                           ****************:**:*:**:* .***************:*:: * *.*::* ***

P. oryzae Pro1             RHRYATISALCDALNRDENHQQILEAALGLIFFQCGVGRFDDALPDIAWHQHFQAAISLV
S. macrospora Pro-1        RHRYATITWLCEALNRDENHQPILDATLGLIFFQCIVGRPEDTLPDIPWHQHFQAVVSLV
N. crassa Adv-1            RHRYATITWLCEALNRDENHQPILDATLGLIFFQCIVGRPEDTLPDIPWHQHFQAVVSLV
                           *******: **:********* **:*:******** *** :*:****.*******.:***

P. oryzae Pro1             QKLDLPRIVSDPNAEAAPTPFNMTLTAWIDILGATMQGRAPTFAHTYREKHLSATNSSLG
S. macrospora Pro-1        QKLDLAGLVSDLAKPLAHTPFNMTLTSWIDILGATMLGSSPMFAHTYRHKHLSVNNHSLG
N. crassa Adv-1            QKLDLAGLVSDITKPLAHTPFNMTLTSWIDILGATMLGSSPLFAHTYRNKHLSINNHSLG
                           *****. :***     * ********:********* * :* ****** **** .* ***

P. oryzae Pro1             LRELMGCEDRVMYLISEIACLEALRNDGMDDITLCQHVHVLGDQIGLTEVGETAPKLPFN
S. macrospora Pro-1        LRELMGCEDRVMYLISEIACLESLKNQGMDDITLCQHVRGLGDEISNTEINEGTLVEPYN
N. crassa Adv-1            LRELMGCEDRVMYLISEIACLESLKNQGMDDITLCQHVRGLGDEISNTEVNEGTLVEPYN
                           **********************:*.*:***********. ***:*. **:.* :   *:*

P. oryzae Pro1             SNGSLSPKQLSKNITAAFRLAARIYLCSLVPGFNPTQASCIGLVEKLTNVLQHIPSGHQG
S. macrospora Pro-1        ANGTLSPKQLSKNITAAFRLAARIYLCSLVPGFHPAQPSCMGLVEKLTAVLQLIPSGVNG
N. crassa Adv-1            ANGTLSPKQLSKNITAAFRLAARIYLCSLVPGFYPAQPSCMGLVEKLTAVLQLIPSGVNG
                           :**:***************************** *:*.**:******* *** **** :*

P. oryzae Pro1             FDRSLVWVYLVGGSVSQPGSSFRGFFEDRVAQLGEAANFGSFGRVSSLLRETWTEADQLV
S. macrospora Pro-1        YDRSLTWVYLIGGSVSVDGSSFRDFFETRIAQLGDAASSGSFGRTTVLLRETWVQYAAAV
N. crassa Adv-1            YDRSLTWVYLIGGSVSVPGSSFRDFFENRVAQLGDVANSGSFGRMTVLLREVWLQYEAIL
                           :****.****:*****  *****.*** *:****:.*. ***** : ****.* :    :

P. oryzae Pro1             AMQSQACSPAASETGSVIPT-----HYIHWRDVMQTKGWDYLLV
S. macrospora Pro-1        AAYE--ASKAAAGNSSPQPQE----PYVRWRDVMQTEGWDYLLI
N. crassa Adv-1            AAAEAAAGTSSLSSETPQQQQQVNAHYVRWREVMQIKGWDYLLI
                           *  .  .. ::  . :          *:.**:*** :******:
```

Fig. S7. Alignment of amino acid sequences of *P. oryzae* Pro1 and its orthologs in *Sordaria macrospora* and *Neurospora crassa*, related to Fig. 3. The Zn(II)$_2$Cys$_6$ DNA-binding motif is shaded in black. Asterisks, double dots, and single dots represent consistent, similar, and dissimilar amino acids among the three species, respectively.

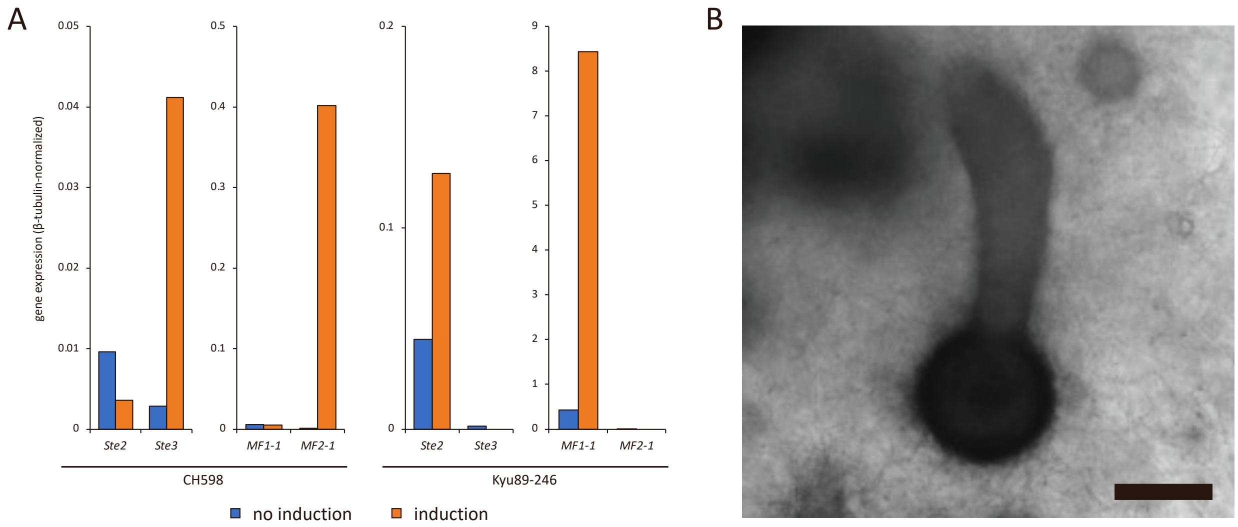

Fig. S8. Induction of sexual reproduction in RY broth, related to Fig. 4. (**A**) The mRNA expressions of the pheromone and receptor genes under sexual reproduction-inducible or non-inducible conditions were quantified by RT-qPCR. CH598 (*MAT-α*) and Kyu89-246 (*MAT-HMG*) were individually cultured on YEG broth for three days at 28°C in the dark (no induction) or on RY broth for seven days at 20°C under fluorescent light (induction). *Ste2* and *MF1-1* correspond to *MAT-HMG* and *Ste3* and *MF2-1* correspond to *MAT-α*. (**B**) Perithecium development was observed in mixed cultures of CH598 and Kyu89-246 on RY broth at 20°C for four weeks under fluorescent light. Scale bar = 100 µm.

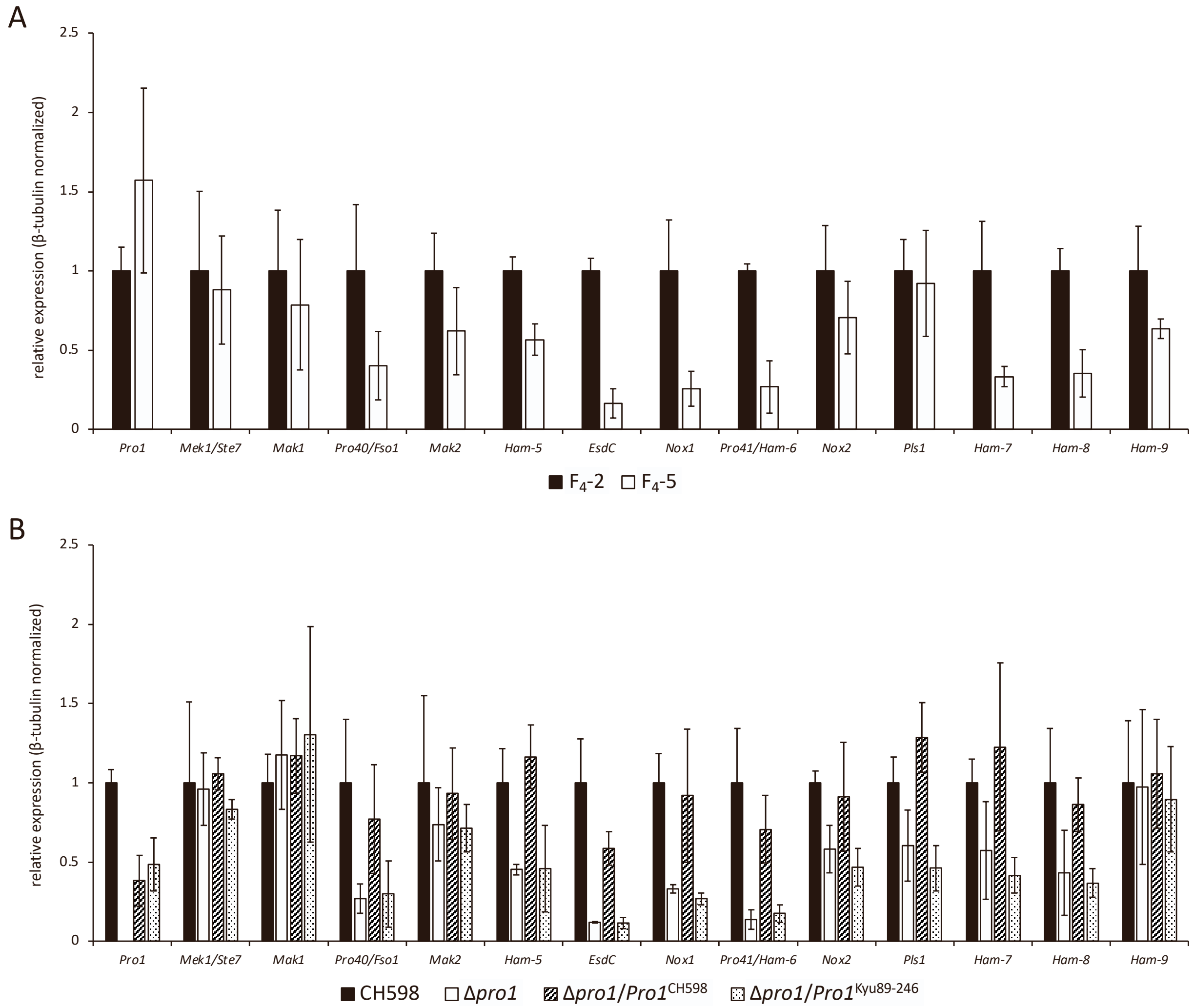

Fig. S9. Expression levels of Pro1-regulated genes confirmed by RT-qPCR, related to Fig. 4. Gene expressions were comparatively quantified by RT-qPCR between (**A**) F_4-2 (female fertile) and F_4-5 (female sterile), and (**B**) CH598 wild type, *Δpro1*, *Δpro1*/*Pro1*^{CH598}, *Δpro1*/*Pro1*$^{Kyu89\text{-}246}$. The experiment was triplicated per strain.

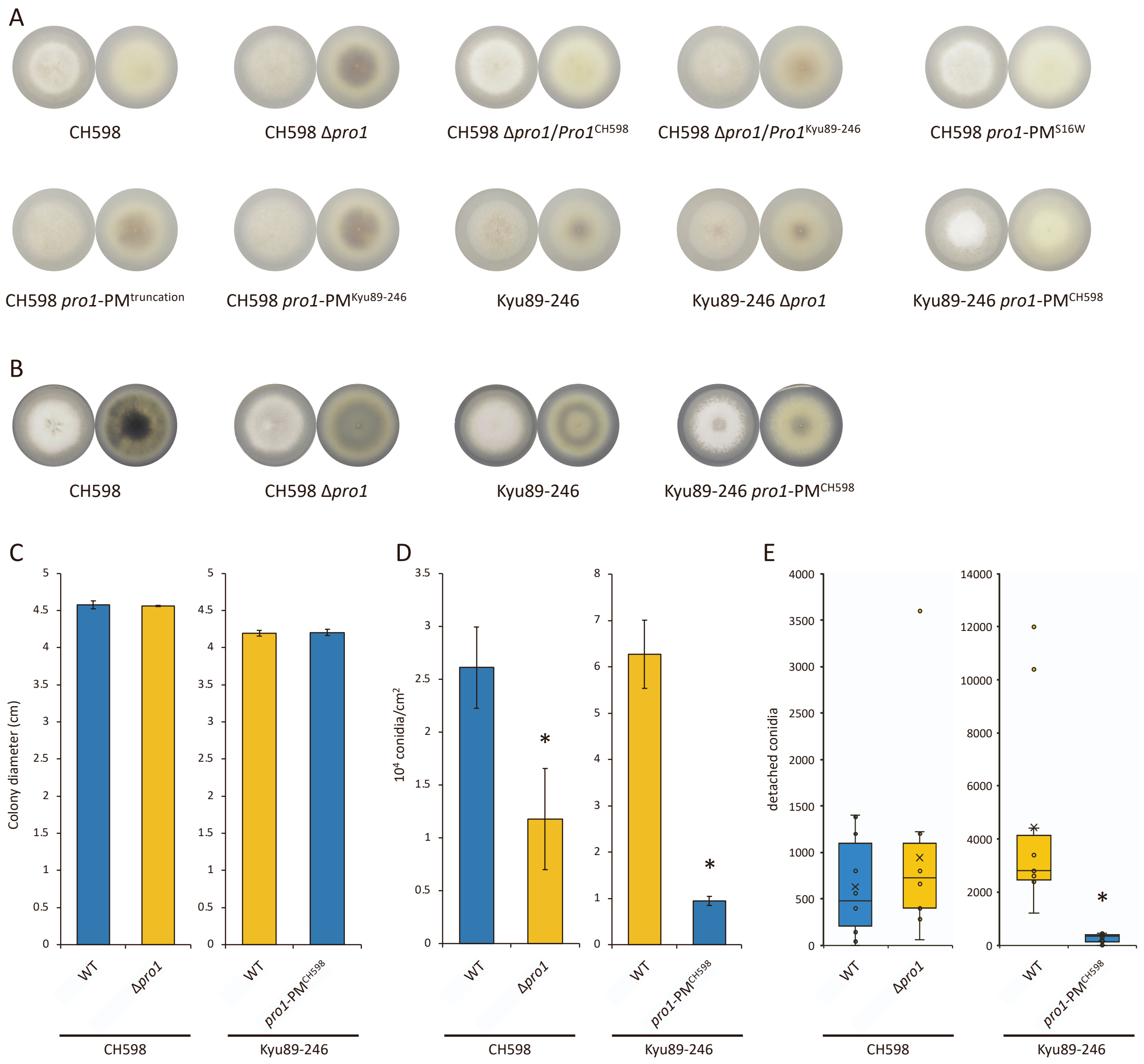

Fig. S10. Phenotypes associated with Pro1 function, related to Fig. 5. Mycelial morphologies of CH598, Kyu89-246, and their transformants grown on (**A**) rice flour medium and (**B**) complete medium (CM) at 28°C for 6 days. Left, from the top; right, from the bottom. (**C**) Colony diameter, (**D**) conidial production, and (**E**) conidial detachment on CM. Bars and error bars in (**C**) and (**D**) indicate the mean ± SEM. Crosses and lines in (**E**) indicate the mean and median, respectively. Blue bars and boxes indicate the results of strains possessing the functional Pro1. Yellow bars and boxes indicate the results of strains possessing the dysfunctional Pro1. * $p < 0.05$ (Welch's *t*-test).

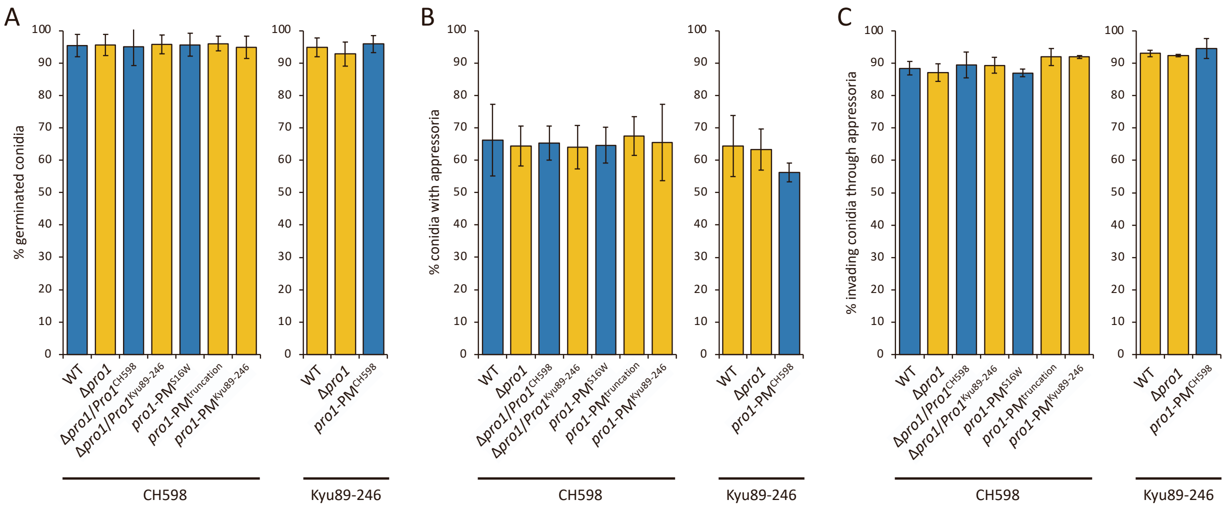

Fig. S11. Asexual phenotype associated with Pro1 function, related to Fig. 5. (**A**) Percentage conidial germination and (**B**) appressorium formation, and (**C**) invasion through appressoria in the CH598 and Kyu89-246 genetic backgrounds. No significant difference was detected in each genotype. Bars and error bars indicate the mean ± SEM. Blue, strains with functional Pro1; yellow, with dysfunctional Pro1.

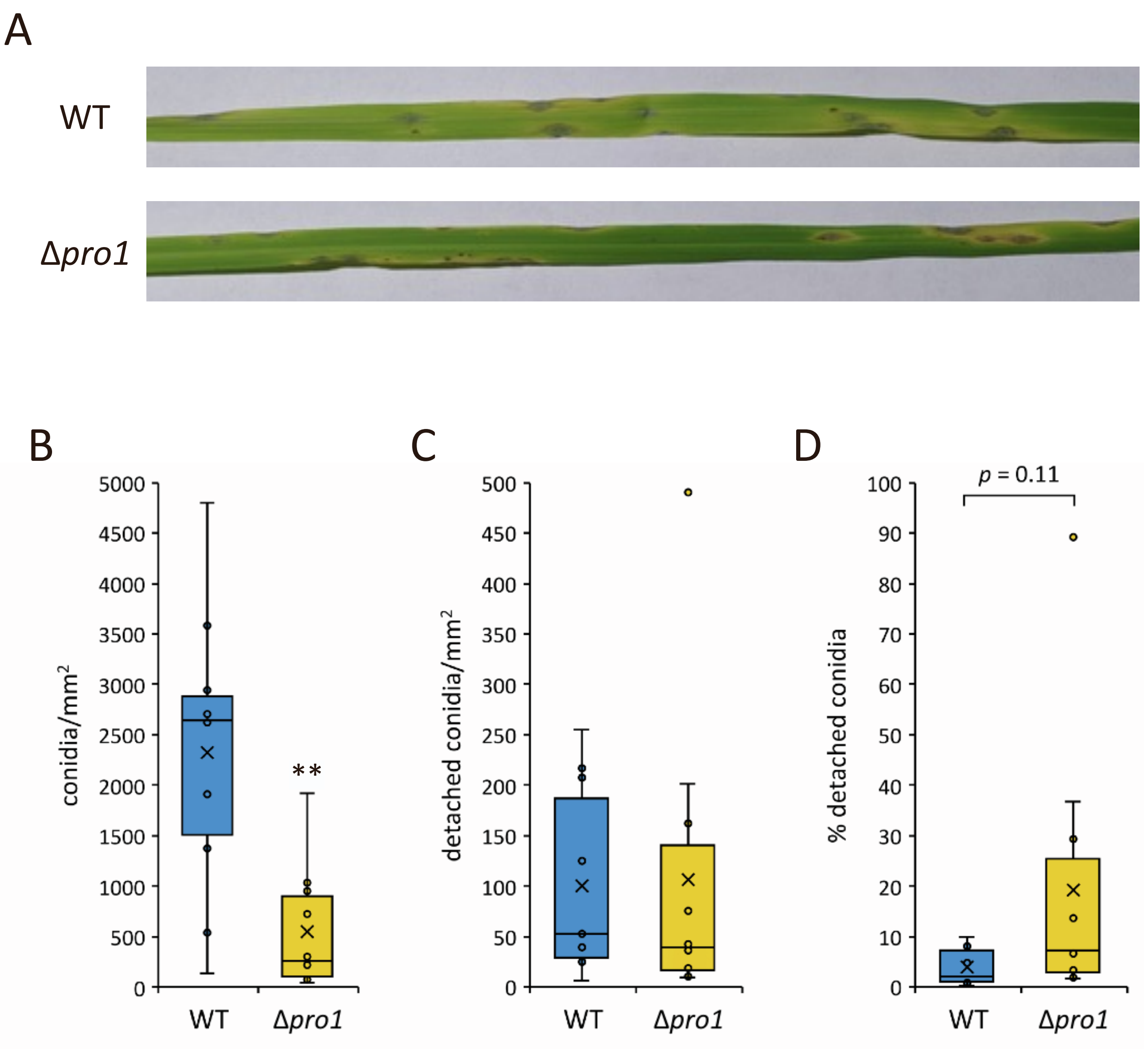

Fig. S12. Asexual phenotypes observed on rice plants, related to Fig. 5. (**A**) Lesions formed by CH598 wild type (upper) and Δ*pro1* mutant (lower) on the rice leaves of Nipponbare. (**B**) Box plots indicate the number of conidia produced per square millimeter of lesion. (**C**) Box plots indicate the number of conidia detached per square millimeter of lesion. Experiments in (**B**) and (**C**) were performed with ten lesions. ** $p < 0.01$ (Welch's *t*-test, compared to the wild type).

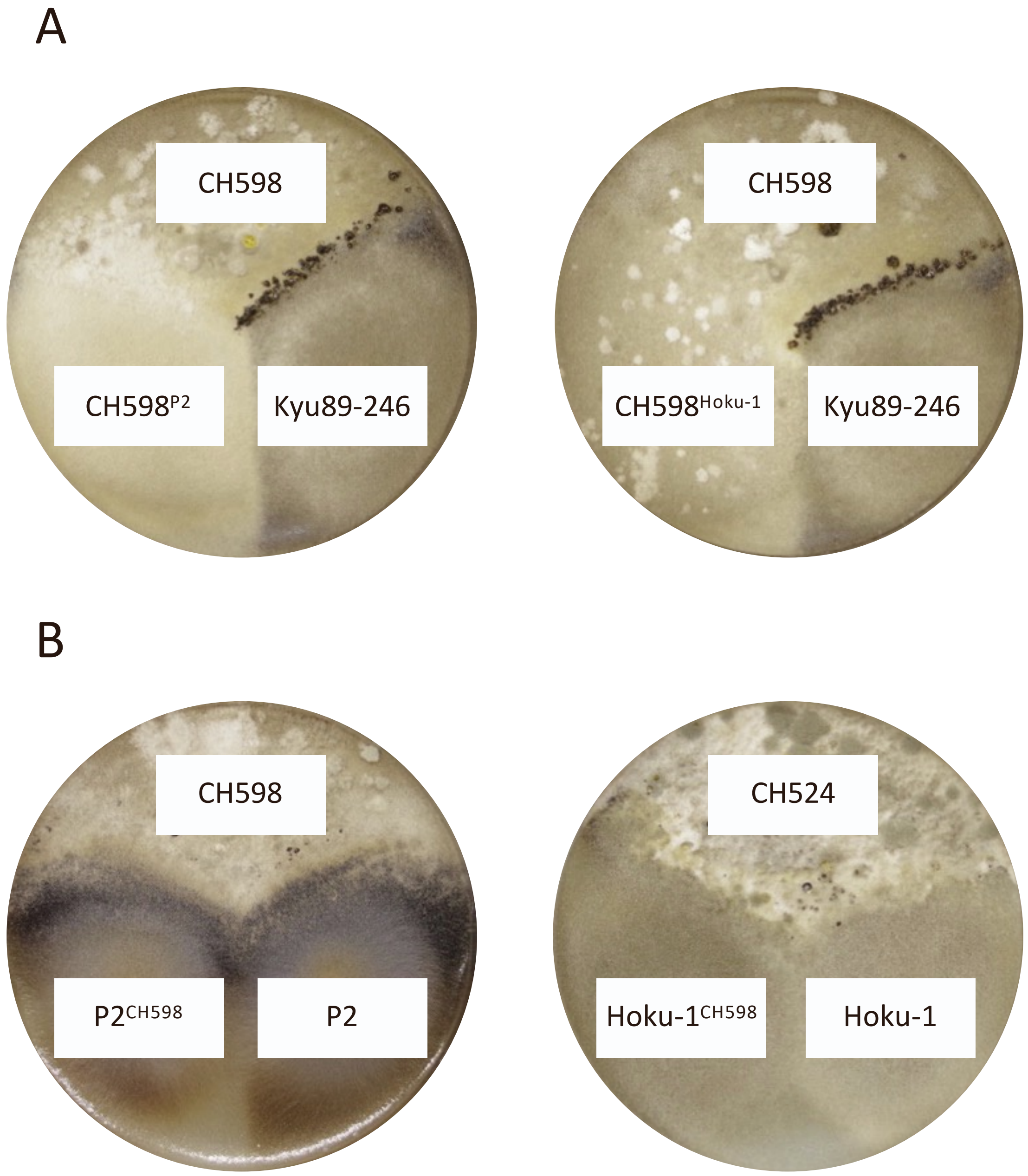

Fig. S13. Functions of mutated Pro1 in P2 and Hoku-1 as determined by crossing, related to Fig. 7. (**A**) CH598 mutants possessing P2- or Hoku-1-derived *Pro1* variants did not develop perithecia. (**B**) Functional *Pro1* integrated into P2 and Hoku-1 did not rescue their fertility. Superscripts represent the sequence origin of *Pro1*.

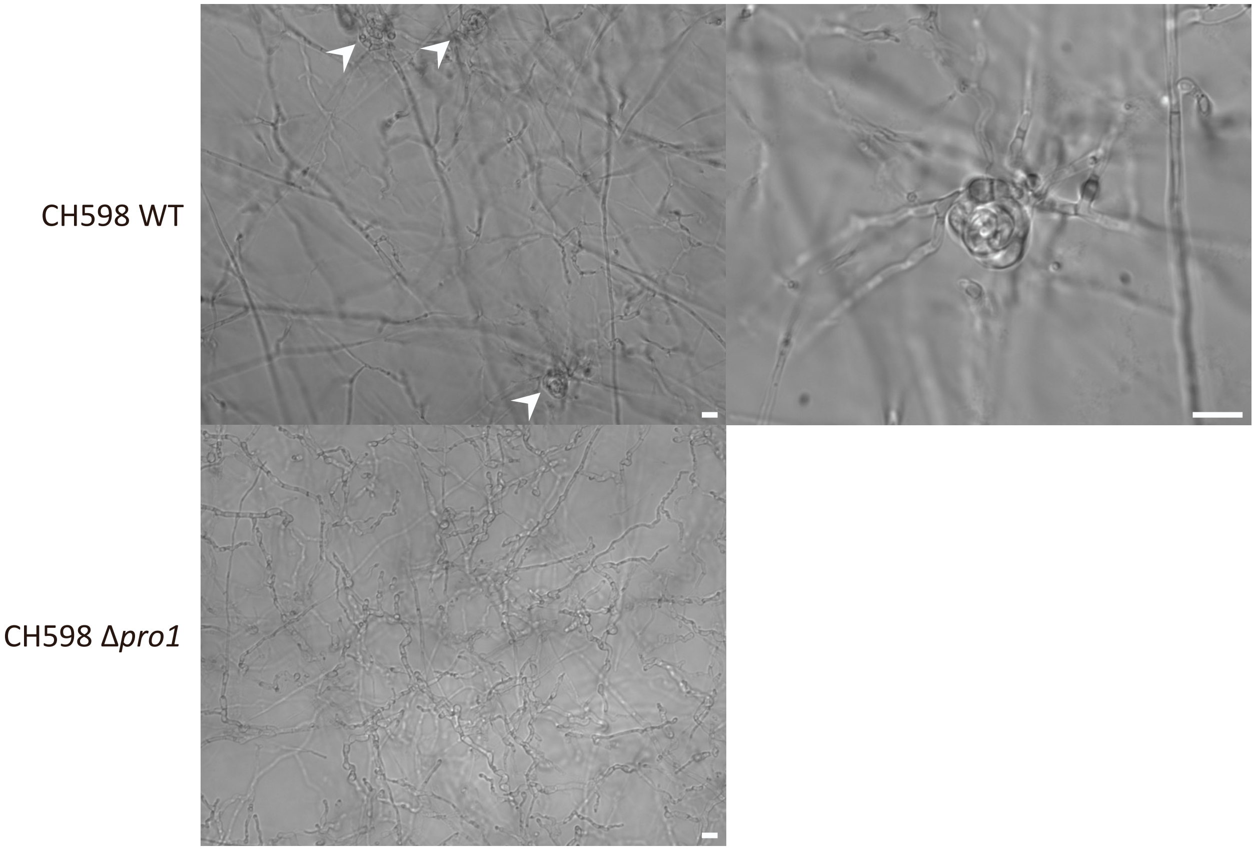

Fig. S14. Ascogonium development, related to Fig. 3. CH598 or Δ*pro1* was individually cultured on RY broth at 20°C for three weeks under fluorescent light. CH598 often developed ascogonia (upper, arrowed), while the structures were not observed in Δ*pro1* (lower). Scale bar = 10 μm.

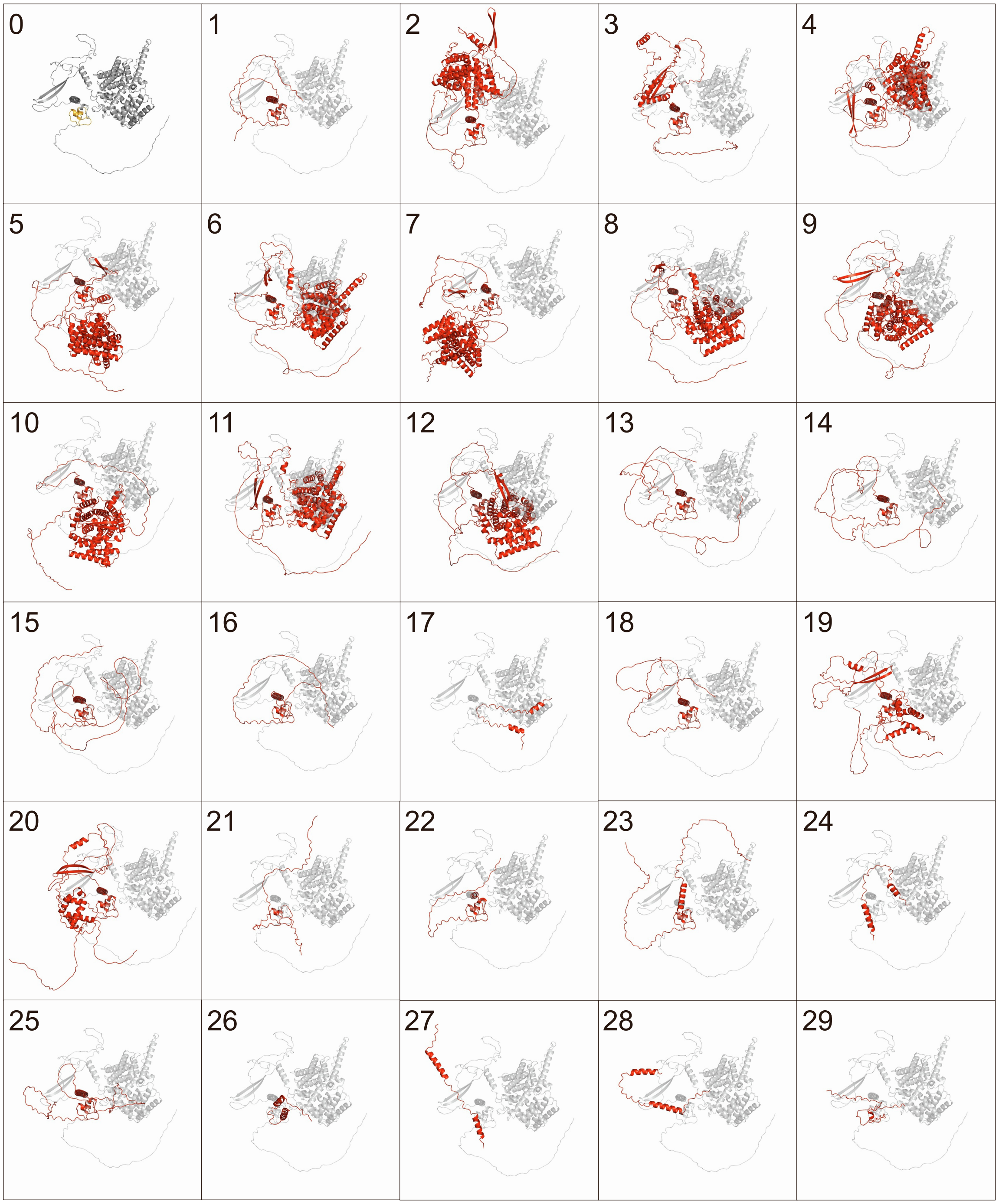

Fig. S15. Predicted protein structures of Pro1 variants, related to Fig. 7. The rank_1 predictions of protein folding by ColabFold are shown. The domain colored yellow in variant #0 is the Zn(II)$_2$Cys$_6$ DNA-binding domain. Protein structures for each variant are colored red, together with variant #0, colored light grey. Structures were aligned at the DNA-binding domain, if possible, or oriented most plausibly.

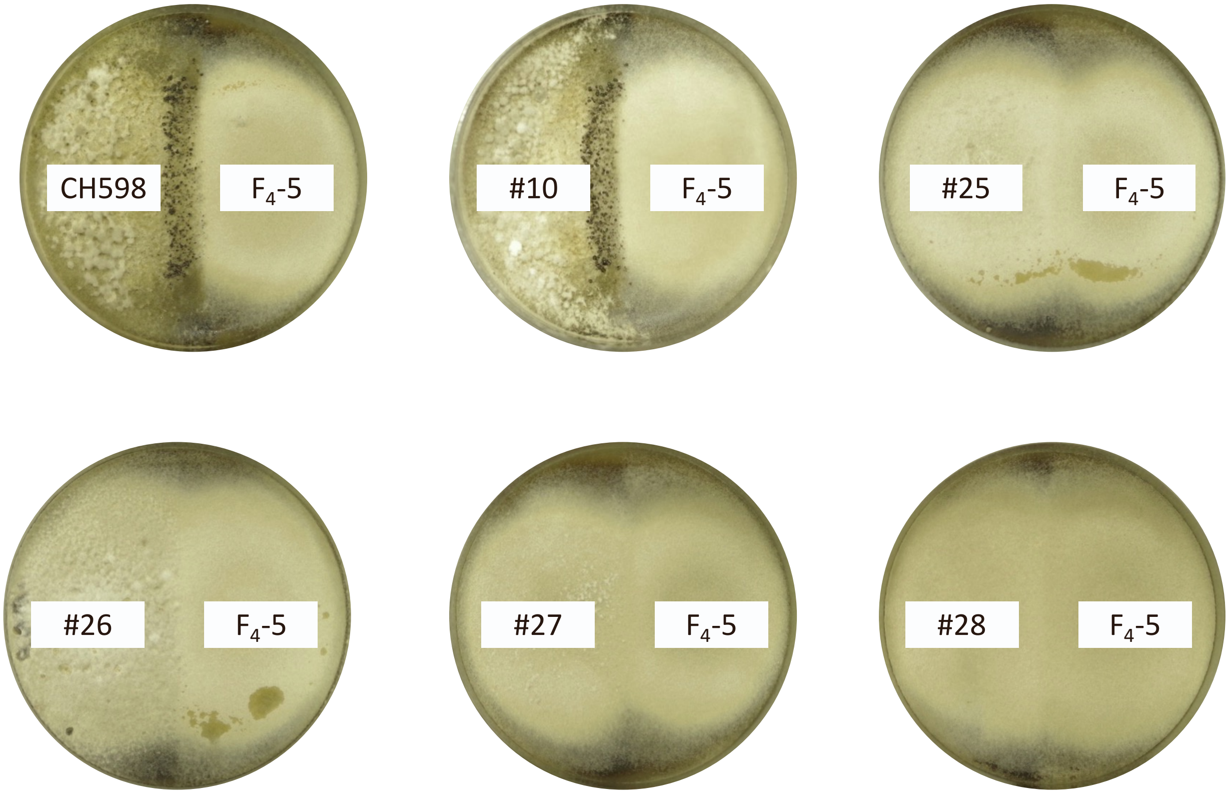

Fig. S16. Functions of some Pro1 variants, related to Fig. 7. CH598 mutants possessing five *Pro1* variants (#10 and #25-28) were tested. While the variant #10 (single amino acid substitution) retained function in female fertility, the other variants with truncation affect perithecium development.

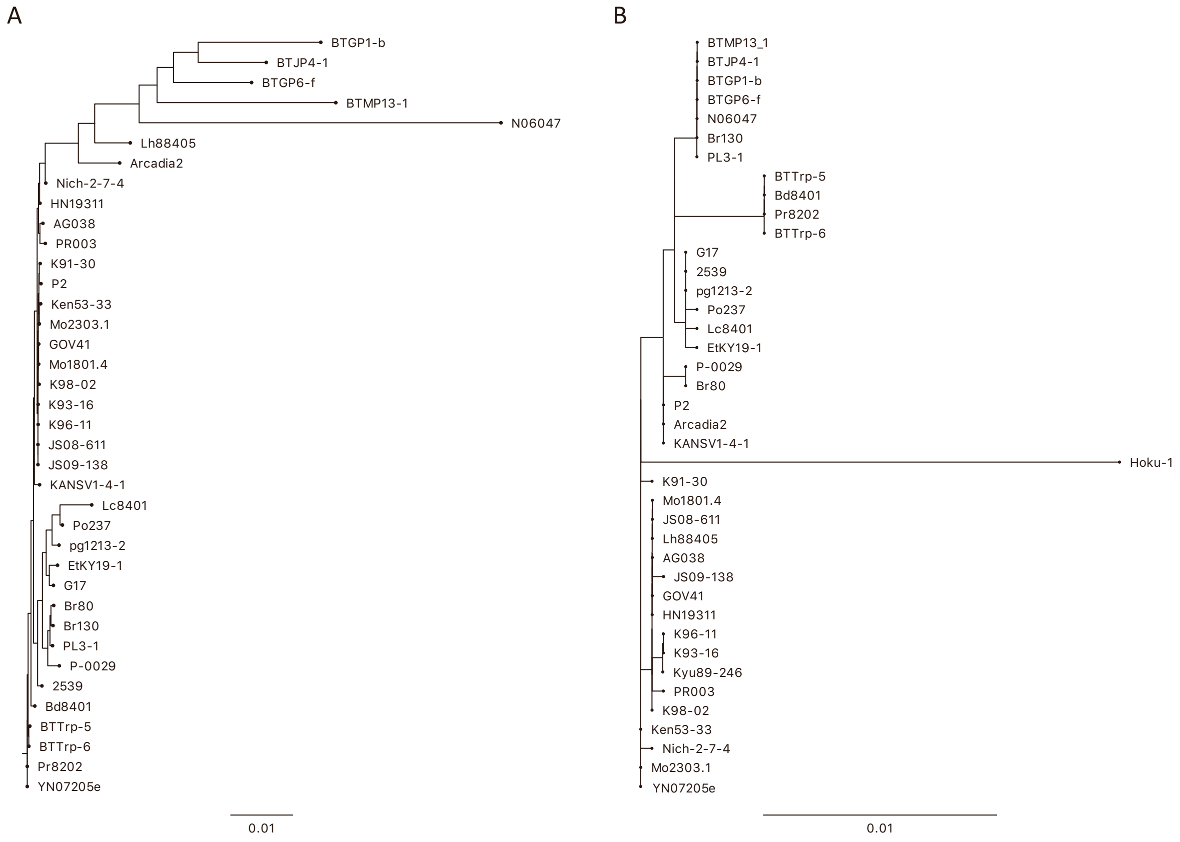

Fig. S17. Phylogenomic and *Pro1* phylogenetic trees, related to Fig. 7. (**A**) The phylogenomic tree of 38 isolates drawn by nucleotide sequences of 468 BUSCO genes, which were detected in all strains as single copies. (**B**) The phylogenetic tree of *Pro1* ORF sequences from the 40 isolates possessing Pro1 mutations. Kyu89-246 and Hoku-1 are missing in the phylogenomic tree because of no available genomic sequence. YN07205e, a Yunnan (the origin of *P. oryzae*) isolate possessing functional Pro1.

Table S1. Segregation of the mating type and female fertility in F_4 progenies, related to Fig. 1. The numbers of strains for each genotype or phenotype are shown. Numbers in Parentheses are the values when F_4-4, which is thought to be a putative female-sterile-evolved strain because of inconsistent genotype through FS1 region, is treated as a female-fertile strain.

	female fertile	female sterile	total	χ^2-test (1:1)	χ^2-test (1:3)
MAT1-a	6 (7)	10 (9)	16	χ^2 = 0.68<br>*p* = 0.41	χ^2 = 6.57<br>*p* = 0.01
MAT1-HMG	10	11	21		
total	16 (17)	21 (20)	37		
χ^2-test (1:1)	χ^2 = 0.68 (0.24)<br>*p* = 0.41 (0.62)				
χ^2-test (1:3)	χ^2 = 6.57 (8.66)<br>*p* = 0.01 (0.00)				
